# A Neonatal Case of Lower Urinary Tract Obstruction and Imperforate Anus: Urethral Decompression Due to Rectourinary Fistula

**DOI:** 10.7759/cureus.60404

**Published:** 2024-05-16

**Authors:** Shotaro Sugita, Kentaro Tamura, Mitsuhide Nagaoka, Katsuhisa Hirano, Taketoshi Yoshida

**Affiliations:** 1 Division of Neonatology, Maternal and Perinatal Center, Toyama University Hospital, Toyama, JPN; 2 Department of Surgery and Science, Faculty of Medicine, Academic Assembly, University of Toyama, Toyama, JPN

**Keywords:** neonate, lower urinary tract obstruction, imperforate anus, enteroliths, chronic renal failure

## Abstract

Lower urinary tract obstruction (LUTO) is a rare fetal condition associated with significant perinatal morbidity and mortality. Herein, we report a neonatal case of LUTO with anal atresia complicated by anhydramnios and pulmonary hypoplasia. After treatment for severe postnatal respiratory distress, the neonate underwent vesicostomy and colostomy. Postoperatively, respiratory status and renal function improved. This case highlights a unique feature where a large rectovesical fistula channeled fetal urine into the colon, which minimized obstructive damage to the urinary tract and preserved renal morphology. Fetal colonic dilatation and numerous enteroliths indicate urine influx into the intestinal tract. Our case suggests the importance of recognizing such exceptions in complete LUTO to predict postnatal outcomes diagnosed in utero.

## Introduction

Lower urinary tract obstruction (LUTO) is a rare fetal condition characterized by the partial or complete obstruction of the bladder and urethra [1,2]. Complete LUTO significantly increases perinatal morbidity and mortality caused by two pathologies. First, the inability to void urine leads to oligohydramnios, which is linked to pulmonary hypoplasia [1]. Second, fetal urine retention increases the pressure in the bladder and upper urinary tract, causing renal and bladder dysfunction. Despite surviving the initial postnatal respiratory problems, end-stage renal failure may still develop, requiring pediatric dialysis and kidney transplantation [3,4].

This study presents a neonatal case of LUTO with anal atresia associated with anhydramnios and pulmonary hypoplasia. He had a dilated intestinal tract with numerous enteroliths and a thickened rectourinary fistula. Notably, despite having complete LUTO, the neonate exhibited preserved renal function. We explored the factors contributing to this unique presentation.

## Case presentation

**Figure 1:**
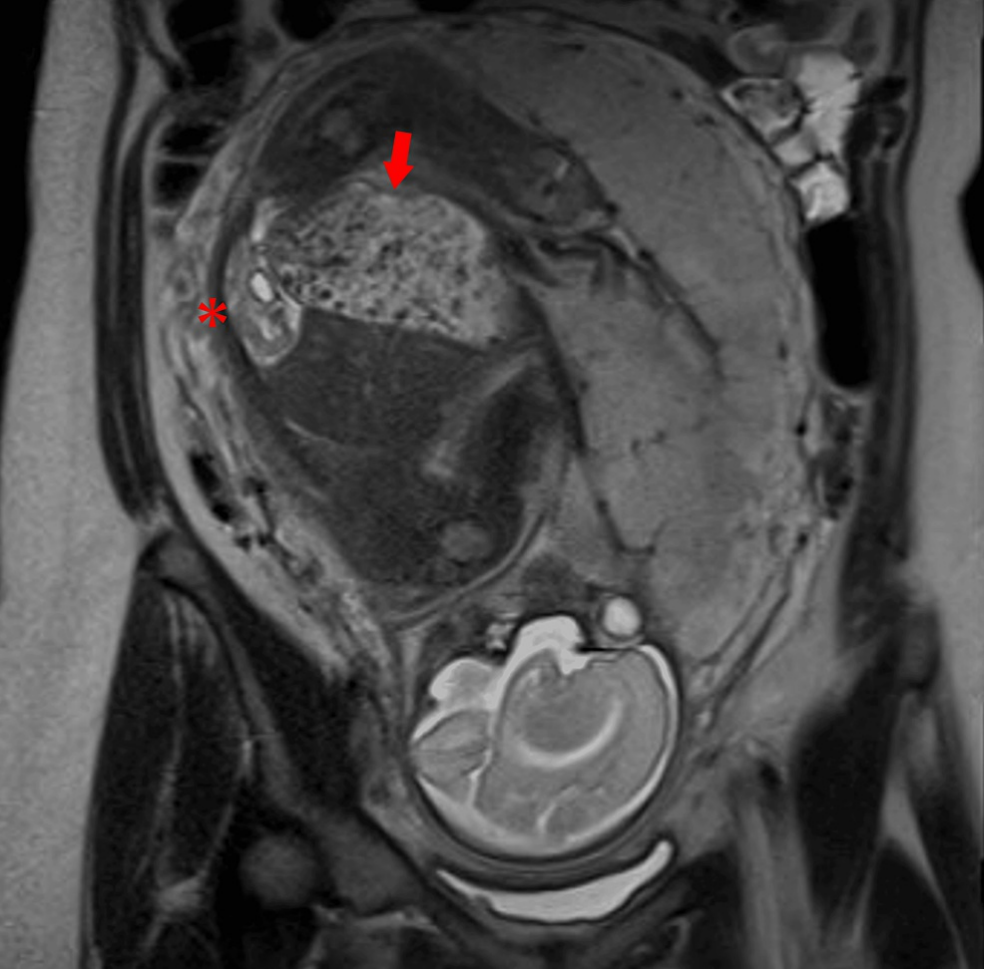
Fetal magnetic resonance imaging at 29 weeks of gestation Fetal magnetic resonance imaging showing anhydramnios and intestinal dilation containing low signal intensity nodules (arrow). Kidney sizes are normal (*).

The mother, a 26-year-old woman, gravida 2, para 1, was referred to our institution at 17 weeks of gestation for fetal abdominal cyst and anhydramnios. A fetal magnetic resonance image at 29 weeks of gestation showed dilation from the descending colon to the rectum, containing nodules of low signal intensity. No amniotic fluid and no bladder dilation were observed. Although mild bilateral renal pelvic dilation was noted, the kidneys had normal sizes (Figure 1).

**Figure 2:**
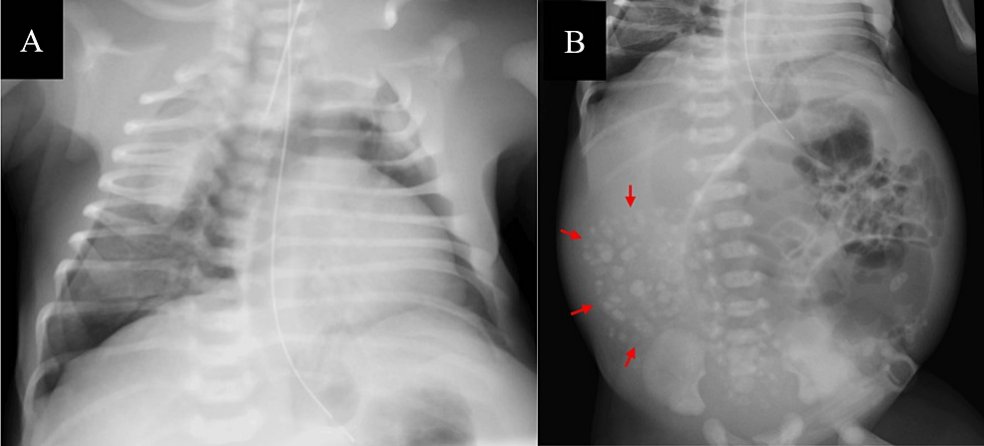
Initial radiographs of the patient (A) Chest X-ray image showing bilateral tension pneumothorax. (B) Abdominal X-ray image showing an enlarged intestinal tract and numerous calcifications in the lower abdomen (arrows).

At 32 weeks of gestation, the mother went into labor and delivered a male neonate. The neonate was immediately intubated. Birth weight was 2,386 g, with Apgar scores of 3 (1 minute) and 5 (5 minutes). The neonate had severe respiratory distress, and chest X-ray imaging revealed bilateral tension pneumothorax (Figure 2A), prompting the insertion of bilateral chest tubes. Abdominal X-ray imaging revealed gastrointestinal tract enlargement and multiple calcifications in the lower abdomen (Figure 2B).

**Figure 3:**
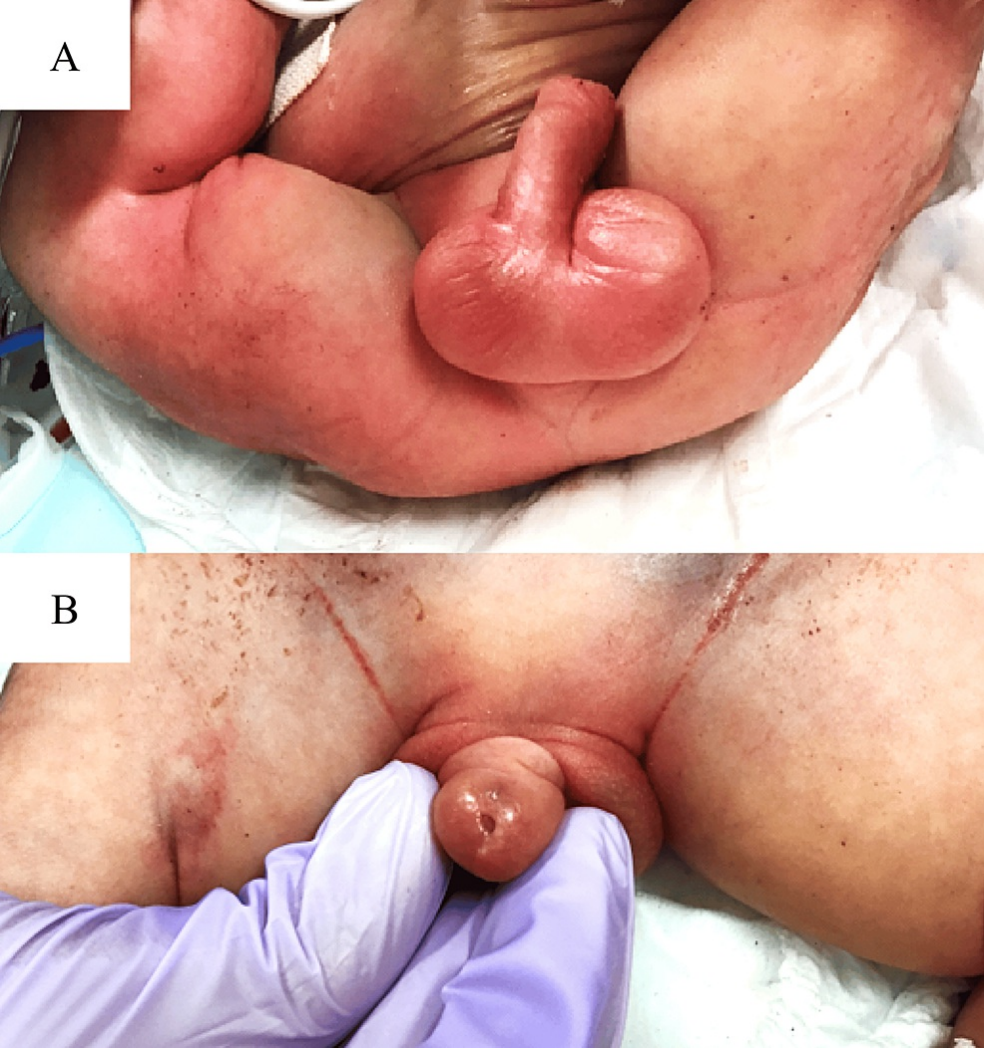
Physical examination of the patient (A) The external genitalia was male with bilateral testis. (B) The external urethral orifice was visible; however, the urinary catheter could not be inserted.

On physical examination, a distended abdomen with an imperforate anus was found (Figure 3A). The external genitalia were male with palpable testis. Although the external urethral orifice was visible, a thin urinary catheter could only reach 5 cm, and urine was absent (Figure 3B).

**Figure 4:**
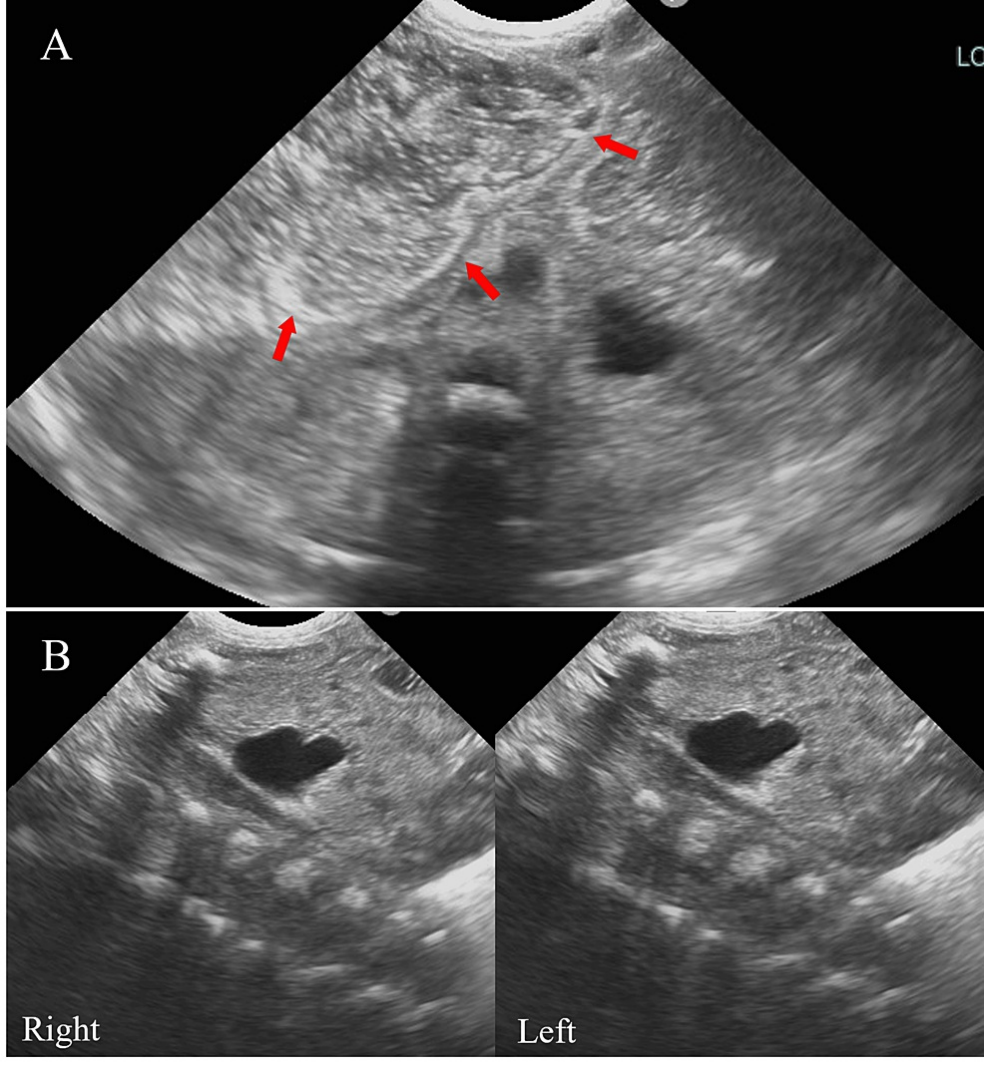
Ultrasonography of the abdomen (A) Image showing numerous small hyperintense nodules with acoustic shadows in the dilated intestinal tract (arrows). (B) Despite the presence of mild bilateral hydronephrosis, the longitudinal diameter of the kidneys is normal.

Ultrasonography revealed a dilated intestinal tract containing numerous small hyperintense images with acoustic shadows (Figure 4A). The longitudinal diameter of the kidneys was normal: 45 and 40 mm on the right and left sides, respectively. Mild bilateral hydronephrosis without hydroureter was found (Figure 4B).

The chromosomal analysis revealed a normal male karyotype (46, XY).

**Figure 5:**
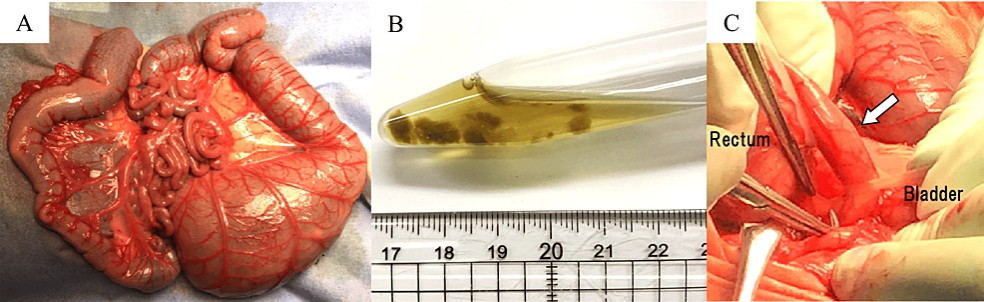
Perioperative photograph (A) Dilation is observed from the rectum to the transverse colon. (B) The intestinal tract is filled with yellow fluid and small enteroliths. (C) A rectovesical fistula is observed between the bladder neck and the rectum (arrow).

Respiratory status was unpredictable, necessitating several treatment approaches, including ventilatory management, nitric oxide inhalation therapy, and high-frequency oscillatory ventilation. At two days old, he was still unable to urinate, and his serum creatinine level increased to 1.7 mg/dL, prompting a percutaneous cystocentesis procedure in the neonatal intensive care unit (NICU). At four days old, his respiratory status gradually improved, and cutaneous vesicostomy and colostomy were performed under general anesthesia. Severe dilatation was observed from the rectum to the transverse colon (Figure 5A). The intestinal tract was filled with yellow fluid and 2-5 mm large enteroliths (Figure 5B). A large rectovesical fistula was identified between the bladder neck and the rectum (Figure 5C). The fistula was ligated.

The postoperative course was uneventful, and the infant was weaned from ventilatory support at 16 days old. Complete weaning from ventilatory support was achieved at 52 days. He urinated continuously through the vesicostomy, and the serum creatinine level decreased to 0.26 mg/dL. After establishing stable oral feeding, he was discharged from the NICU at four months old. At six months, his body weight and height were 5.2 kg and 58.0 cm, respectively. His renal function remained well-preserved. Anoplasty and urethroplasty will be performed in the future.

## Discussion

This is a case of a neonate who survived with complete LUTO without severe renal dysfunction. Fetal urine is drained to the colon through a large recto-bladder neck fistula, minimizing obstructive damage to the urinary tract. Intestinal dilatation and numerous enteroliths indicated the influx of fetal urine into the intestinal tract.

Fetal intraintestinal meconium calcification is rare, and echogenic foci within a dilated fetal colon may indicate a rectourinary fistula [5,6]. The exact mechanism of intraluminal meconium calcification remains unclear. Shimotake et al., using infrared spectroscopic analysis, suggested that luminal fecal calcium was derived from fetal feces and fetal urine [7]. Meconium comprises putrefactive intestinal wall products and concentrated bile salts and is high in calcium. Studies have suggested that pH changes may precipitate calcium salts [7]. Presumably, urine engulfs the meconium, forming a spherical conformation around the calcium aggregates [8].

A posterior urethral valve, followed by urethral atresia or stenosis, was reported as the most common cause of LUTO [1]. Suspicion of LUTO arises from fetal ultrasound findings such as dilated bladder, dilated posterior urethral diameter, thickened bladder wall, dilated ureters, oligohydramnios/anhydramnios, and abnormal renal parenchyma [1,9,10]. Complete LUTO causes lower and upper urinary tract obstruction, resulting in impaired kidney development and bladder dysfunction [11]. Renal dysplasia causes hypoplasia with decreased nephron volume, which can lead to chronic kidney disease (CKD), progressing to end-stage renal disease [3,4]. Bladder dysfunction is exacerbated by polyuria in the affected kidney and can lead to secondary vesicoureteral reflux and recurrent urinary tract infections. This condition causes scarring of the renal parenchyma and accelerates CKD progression [3,4].

In this case, anhydramnios caused by complete LUTO was observed, leading to lung hypoplasia. Consequently, postnatal respiratory management was difficult, but it improved within a few days of birth. Advances in neonatal medicine have led to reported cases of survival even with renal oligohydramnios [12,13]. A giant bladder or dilated ureter is common in complete LUTO [1]. Despite reports of the effectiveness of fetal treatment with a bladder-amniotic fluid shunt [14], there was no dilatation of the urinary tract in our case. Instead, renal morphology was preserved by the decompression from the rectourinary fistula, potentially preventing obstructive renal damage. We will continue to evaluate renal and bladder function while taking measures to prevent vesicoureteral reflux, urinary tract infections, and overactive bladder syndrome [15]. In the future, we also plan to perform urethroplasty and repair the imperforate anus.

## Conclusions

This case highlights a complete LUTO survivor who escaped severe renal dysfunction. Fetal urine draining into the colon through a rectourinary fistula minimized obstructive kidney and urinary tract damage. This case may provide useful information for predicting postnatal prognosis in LUTO cases diagnosed in utero. Complete LUTO may be survivable in cases with preserved renal morphology without urinary tract dilation and if the patient survives postnatal respiratory failure.

## References

[1] Cheung KW, Morris RK, Kilby MD (2019). Congenital urinary tract obstruction. Best Pract Res Clin Obstet Gynaecol.

[2] Taghavi K, Sharpe C, Stringer MD (2017). Fetal megacystis: a systematic review. J Pediatr Urol.

[3] Freedman AL, Johnson MP, Smith CA, Gonzalez R, Evans MI (1999). Long-term outcome in children after antenatal intervention for obstructive uropathies. Lancet.

[4] Holmes N, Harrison MR, Baskin LS (2001). Fetal surgery for posterior urethral valves: long-term postnatal outcomes. Pediatrics.

[5] Pohl-Schickinger A, Henrich W, Degenhardt P, Bassir C, Hüseman D (2006). Echogenic foci in the dilated fetal colon may be associated with the presence of a rectourinary fistula. Ultrasound Obstet Gynecol.

[6] Shiozaki A, Yoneda S, Iizuka T (2015). Prenatal diagnosis of enterolithiasis at 18 weeks: multiple foci of intraluminal calcified meconium within echogenic bowel. J Med Ultrason (2001).

[7] Shimotake T, Higuchi K, Tsuda T, Aoi S, Iwai N (2006). Infrared spectrophotometry of intraluminal meconium calculi in a neonate with imperforate anus and rectourethral fistula. J Pediatr Surg.

[8] Berdon WE, Baker DH, Wigger HJ, Mitsudo SM, Williams H, Kaufmann HJ, Shapiro L (1975). Calcified intraluminal meconium in newborn males with imperforate anus: enterolithiasis in the newborn. Am J Roentgenol Radium Ther Nucl Med.

[9] Malin G, Tonks AM, Morris RK, Gardosi J, Kilby MD (2012). Congenital lower urinary tract obstruction: a population-based epidemiological study. BJOG.

[10] Fontanella F, Duin LK, Adama van Scheltema PN (2018). Prenatal diagnosis of LUTO: improving diagnostic accuracy. Ultrasound Obstet Gynecol.

[11] Klaus R, Lange-Sperandio B (2022). Chronic kidney disease in boys with posterior urethral valves—pathogenesis, prognosis and management. Biomedicines.

[12] Mehler K, Beck BB, Kaul I, Rahimi G, Hoppe B, Kribs A (2011). Respiratory and general outcome in neonates with renal oligohydramnios—a single-centre experience. Nephrol Dial Transplant.

[13] Miyahara J, Yamamoto M, Motoshige K, Fujita N, Ohki S (2016). Survival of a very low-birthweight infant with Potter sequence on long-term hemodialysis. Pediatr Int.

[14] Kanamori Y, Iwanaka T, Nakahara S (2008). Survival in a neonate with complete urorectal septum malformation sequence after fetal vesico-amniotic shunting for a prominently dilated cloaca. Fetal Diagn Ther.

[15] Adriaenssens M, De Boe V (2023). Congenital lower urinary tract obstruction with spontaneous fetal bladder rupture due to posterior urethral valves: a case report. J Med Case Rep.

